# Genome-wide gain-of-function screen for genes that induce epithelial-to-mesenchymal transition in breast cancer

**DOI:** 10.18632/oncotarget.11314

**Published:** 2016-08-16

**Authors:** Dubravka Škalamera, Mareike Dahmer-Heath, Alexander J. Stevenson, Cletus Pinto, Esha T. Shah, Sheena M. Daignault, Nur Akmarina B.M. Said, Melissa Davis, Nikolas K. Haass, Elizabeth D. Williams, Brett G. Hollier, Erik W. Thompson, Brian Gabrielli, Thomas J. Gonda

**Affiliations:** ^1^ University of Queensland Diamantina Institute, University of Queensland, Translational Research Institute, Brisbane, QLD, Australia; ^2^ St Vincent's Institute of Medical Research and University of Melbourne Department of Surgery, St. Vincent's Hospital, Melbourne, VIC, Australia; ^3^ Australian Prostate Cancer Research Centre-Queensland, Brisbane, QLD, Australia; ^4^ Institute of Health and Biomedical Innovation and School of Biomedical Sciences, Queensland University of Technology, Translational Research Institute, Brisbane, QLD, Australia; ^5^ Monash Institute of Medical Research (now Hudson Institute of Medical Research), Monash University, Melbourne, VIC, Australia; ^6^ University of Malaya, Kuala Lumpur, Malaysia; ^7^ The Walter and Eliza Hall Institute of Medical Research, Melbourne, VIC, Australia; ^8^ School of Pharmacy, University of Queensland, Brisbane, QLD, Australia; ^9^ Mater Medical Research Institute, The University of Queensland, Translational Research Institute, Woolloongabba, Australia

**Keywords:** high-content-screening, epithelial-mesenchymal transition, vimentin, lentiviral vectors, breast cancer

## Abstract

Epithelial to mesenchymal transition (EMT) is a developmental program that has been implicated in progression, metastasis and therapeutic resistance of some carcinomas. To identify genes whose overexpression drives EMT, we screened a lentiviral expression library of 17000 human open reading frames (ORFs) using high-content imaging to quantitate cytoplasmic vimentin. Hits capable of increasing vimentin in the mammary carcinoma-derived cell line MDA-MB-468 were confirmed in the non-tumorigenic breast-epithelial cell line MCF10A. When overexpressed in this model, they increased the rate of cell invasion through Matrigel™, induced mesenchymal marker expression and reduced expression of the epithelial marker E-cadherin. In gene-expression datasets derived from breast cancer patients, the expression of several novel genes correlated with expression of known EMT marker genes, indicating their *in vivo* relevance. As EMT-associated properties are thought to contribute in several ways to cancer progression, genes identified in this study may represent novel targets for anti-cancer therapy.

## INTRODUCTION

Epithelial to mesenchymal transition (EMT) is a complex developmental process by which epithelial cells lose their tight connections and cell polarity and transform into more migratory mesenchymal cells [1]. Epithelial to mesenchymal plasticity (EMP) is described as the ability of cells to undergo EMT and the reverse process, mesenchymal to epithelial transition (MET). EMP is a feature of cancer cells affecting progression, metastasis and therapeutic resistance of some carcinomas [2–4]. Although there may be exceptions [5, 6], EMT is believed to be required for invasion and spread of cancer cells from the primary tumour to distant sites, while the MET is thought to be required for subsequent attachment and proliferation of metastatic cells [3, 7]. The mesenchymal state has also been correlated with cancer stem cell characteristics in breast and other cancers [2, 3]. EMT is accompanied by extensive changes in gene expression profiles that underlie well-defined changes in cell shape, motility and molecular content [1]. Hallmarks of EMT include loss of E-cadherin (CDH1), and increase in N-cadherin (CDH2) and vimentin (VIM). These molecular changes are controlled by a series of often concomitantly expressed transcriptional regulators including Snail (SNAI1), Slug (SNAI2), TWIST1/2 and ZEB1/2, and a host of post-transcriptional mechanisms [2, 3]. In breast cancer, EMT-like molecular changes are particularly prominent in basal-like tumours, which are associated with more invasive disease and poor prognosis [8–10]. In cell culture models and *in vivo*, EMT can be initiated by a range of stimuli including growth factors (EGF, TGFβ and HGF), cytokines, extracellular matrix, and hypoxia [1].

Our aim was to identify novel drivers of EMT in breast cancer to provide a resource for increased understanding of this disease and for potential drug targets. Since many of the EMT regulators have been described, discovering new drivers required an unbiased genome-wide approach. High-throughput functional screening is a powerful technique for interrogating the genome in an unbiased manner. It allows discovery of key molecular components underlying complex cellular processes such as EMT and cancer development. The screening process encompasses automated analysis of cellular phenotypes following either individual gene inactivation or ectopic expression. Several studies reported the effect of gene inactivation by siRNA or drugs in cells induced to undergo EMT *in vitro* [11–13]. This loss-of-function approach identified novel genes necessary for the execution of EMT under the cellular contexts used. However, it also limited the discovery to genes that were expressed in the model cell line and were required for the applied external factor to impose the EMT phenotype. In contrast, gain-of-function screening by ectopic gene expression broadens the search and enables identification of genes that can drive EMT in the absence of external stimuli.

Here we describe a set of novel EMT drivers identified in a near genome-wide gain-of-function screen using lentiviral expression vectors. The screen was performed in the MDA-MB-468 breast cancer cell line, which exhibits epithelial mesenchymal plasticity and can be induced to undergo reversible EMT with EGF or hypoxia *in vitro*. The cellular and molecular phenotypes of this cell line during *in vitro* induced EMT have been extensively characterised [13–16]. To explore the relevance of our hits to breast cancer progression, we used two approaches. First, we used public breast cancer patient-derived data to confirm expression of hit genes in relevant tumours *in vivo*. Second, we demonstrated that the hits are capable of increasing both cellular invasiveness and expression of a mesenchymal gene profile in the non-tumorigenic, breast epithelial cell line MCF10A.

## RESULTS

### Identification and confirmation of potential EMT-inducing genes

**Figure 1:**
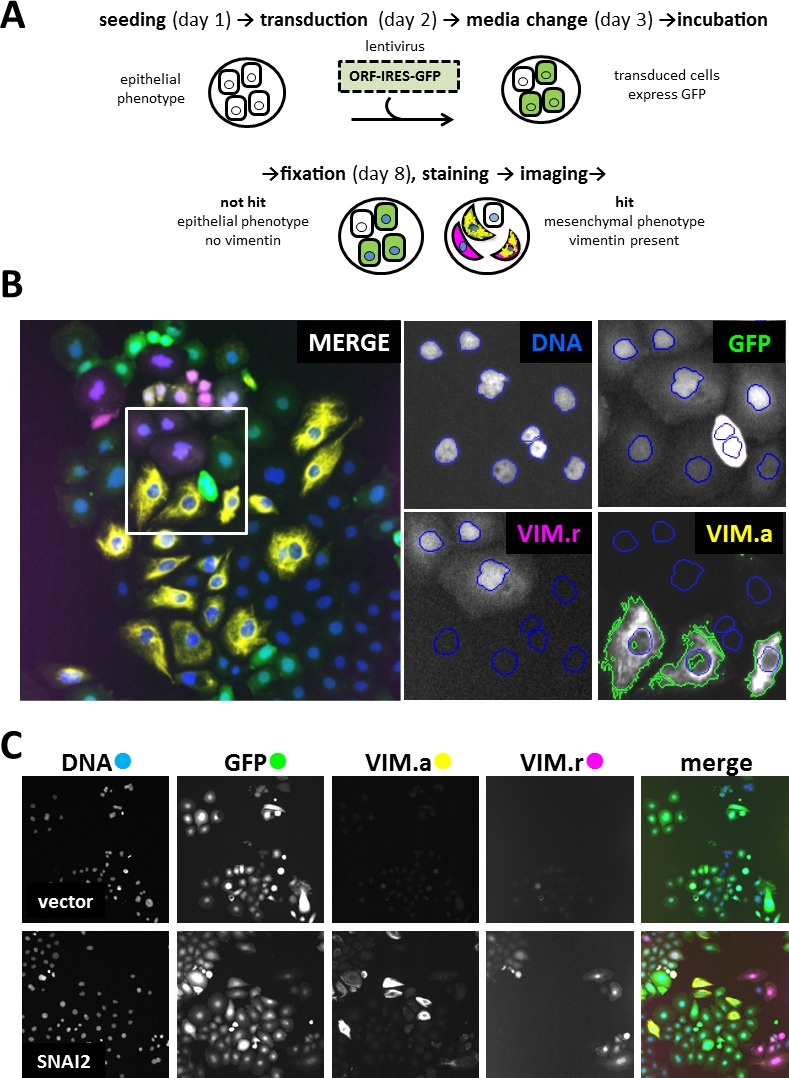
Primary screen assay **A.** Steps in processing 96-well plates during screening. **B.** Image analysis in 4 filter channels. Merged image shows one of the 20 fields of view collected from each well. Four enlarged images indicate mask setting in each channel (Ch1): DNA (Ch1) - DAPI nuclear mask (blue outline) for cells count, GFP (Ch2) - transduced cells selection, VIM.r (Ch3) - dsRed VIM-promoter reporter assay, VIM.a (Ch4) - Alexa647 cytoplasmic anti-vimentin antibody assay. Nuclear mask from Ch1 was extended to the mask of the neighbouring cell, and fluorescence intensity threshold set to select area containing vimentin marker (green outline). Colour of channel name corresponds to pseudocolour in merged overlay image; **C.** Representative field images from control wells: vector (expressing only GFP) as negative control with low VIM signal; *SNAI2* (known inducer of EMT) as positive control with high VIM.a and VIM.r signal. Note that GFP fluorescence indicates presence of the ORF construct, but is not a direct measure of ORF protein levels. All images in C, and overlay in B, 1 microscope field (width = 1321 μm). 20 fields were collected from each well.

To identify novel drivers of EMT, we screened the lentiviral human ORFeome library. Library synthesis and testing have been previously described in detail [17, 18]. The library is arrayed in 96-well plates. Each of the ~17000 wells contains viral supernatant from a lentiviral vector expressing individual human ORF driven by the human EF1-α promoter. The ORFs are co-expressed with IRES-controlled fluorescent marker GFP, allowing for transduced cell identification. The screen was performed on a robot-assisted platform and used automated microscopy and high-content image analysis as the output. To develop a robust and feasible assay, we considered several probes, cell lines and control genes. The mesenchymal marker VIM was chosen because it could be more reliably quantified by automated microscopy compared to the epithelial marker CDH1 or cell shape. CDH1 and cell shape determination required use of additional markers to ascertain plasma membrane localisation and to reliably determine cell boundaries, respectively. Among the breast cancer-derived cell lines tested (PMC42-LA, PMC42-ET, MCF7 and MDA-MB-468), MDA-MB-468 was chosen since it showed highest proportion of transduced cells. In addition, they displayed robustly quantifiable difference in VIM levels between epithelial and mesenchymal states when assayed by automated microscopy. Similar criteria were used for choosing an ORF-expressing positive control virus. Among the supernatants tested (*SNAI1, SNAI2, ZEB1, ZEB2*), *SNAI2* was chosen since it had the strongest effect on VIM levels, and maintained a high proportion of transduced cells without affecting cell viability. The final screening assay for genes capable of inducing EMT is illustrated in Figure 1A. The MDA-MB-468 cell line used contained VIM promoter-construct tagged with red fluorescent protein (dsRed) generated and characterized by Said *et al* [12]. Cells were seeded in microwell plates and grown in medium without EGF, which is normally conducive to an epithelial phenotype and correspondingly little or no VIM expression. They were robotically transduced with lentiviral vectors, incubated for five days, then fixed and prepared for high-content imaging (Figure 1B). DAPI staining allowed cell-nuclei segmentation, while the vector-encoded GFP was used to select transduced cells. VIM expression was quantified both at transcriptional and protein levels, using the red fluorescent VIM promoter-reporter construct (VIM.r) and anti-VIM immunofluorescence (VIM.a) in the Cy5 channel (Figure 1B). Compared to VIM.r, VIM.a allowed better separation between the positive *SNAI2* and negative empty vector controls (Figure 1C). The improved assay sensitivity was indicated by the Z'-factor [19] in test plates which was 0.4 for antibody and - 0.4 for the reporter. Therefore VIM.a was used as the primary assay. Reporter and promoter signal were not always detected in the same cells (Figure 1B, 1C) confirming the well-documented observation that cellular mRNA and protein levels are not always perfectly correlated for a particular gene [20, 21]. In view of this, we have also collected the VIM.r data and used it for additional hit selection.

**Figure 2:**
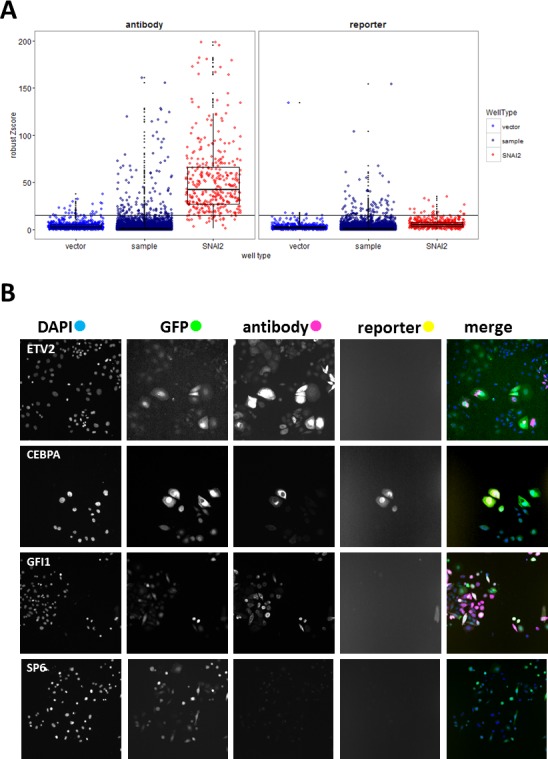
Primary screen analysis **A.** Boxplots (median, 25^th^ and 75^th^ quantile) overlayed with scatterplots comparing normalised VIM fluorescence values (robust Z-scores) between sample and positive (*SNAI2*) and negative (vector) control wells for the antibody and reporter channels. Horizontal black line indicates the hit-picking threshold. Sample images of one of the 20 fields collected per well **B.** from selected hit (*ETV, CEBPA, GFI1*) and non-hit (*SP6*) wells (all images field width = 1321μm).

The complete data from the analysis of 19572 wells (17456 test ORFs and controls) are presented in Table S1. VIM intensity values from 204 plates were normalised using robust Z-scores [22] for sample wells with at least 50 GFP-positive cells (Supplementary Figure S1). Hits were primarily selected using Z-scores (> 15) for VIM.a (Figure 2A, antibody). Proportion of VIM positive cells in vector wells was low (mean = 1.7%, SD = 2.5%), so we selected additional hits from sample wells with any of the following: VIM.r scores above 15 (Figure 2A, reporter), raw intensity values for either reporter or antibody above 8000, or more than 18% of GFP-positive cells that were also VIM-positive. Since this was a first pass screen, the hit criteria were inclusive rather than stringent. This yielded 211 putative hits, and allowed for 4.6% false discovery rate (i.e. of the 814 vector wells 38 fit hit criteria; while of the 411 positive control wells, 19 did not fit hit criteria). Inspection of selected images (Figure 2B) confirmed that high VIM scores corresponded to cells overexpressing VIM. Control wells with false hit status assignment were found to be due to imaging errors (high background, fluorescent debris), rather than changes in VIM expression. As was observed during the assay development, there was no significant correlation between antibody and reporter scores. Only 14 out of 211 putative hits had high values for both, and *SNAI2* control wells were generally high for VIM.a but not for VIM.r (Supplementary Figure S2). VIM levels measured either way did not correlate with cell number or transduction rate (Supplementary Figure S2), indicating that viral titre or potential ORF effects on cell proliferation did not affect hit selection. Our data clearly indicated that cellular VIM protein levels and promoter activity can be independently regulated. As our aim was to identify drivers of EMT, we wanted to focus on genes that can increase VIM protein levels, as well as to avoid technical issues with the VIM promoter-reporter construct, such as transgene loss or silencing and low reporter fluorescence. Therefore, a validation screen was performed using anti-VIM antibody.

**Figure 3:**
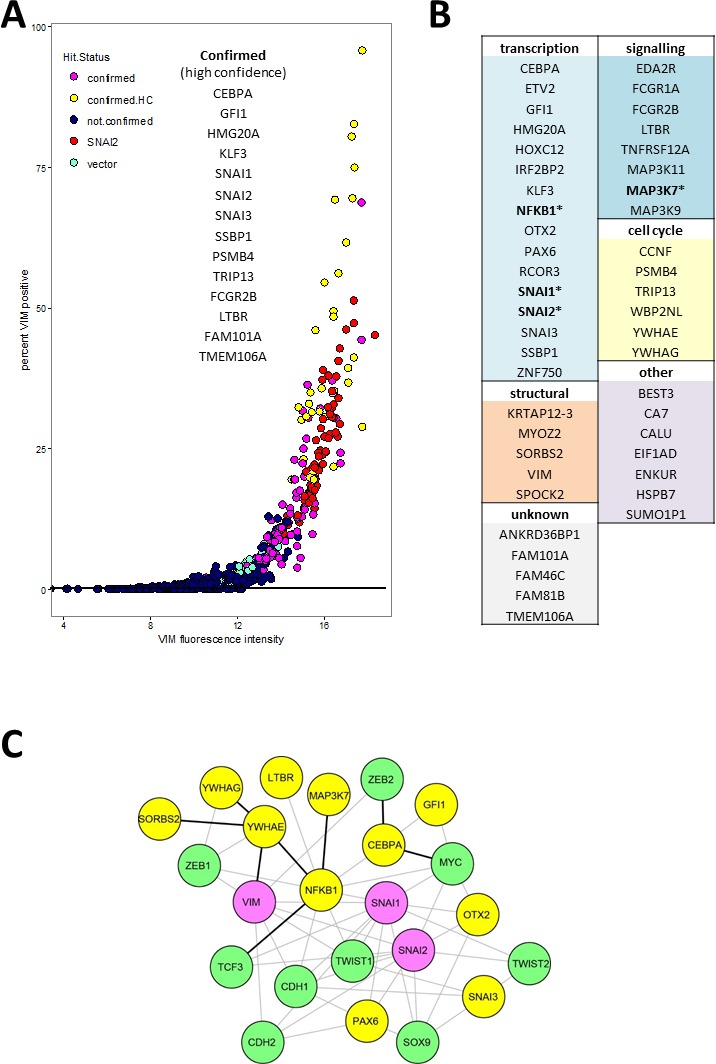
Validated hits **A.** Secondary screen VIM.a total fluorescence intensity (x-axis) and percentage of VIM positive cells. Each point represents data from a single well. Confirmed high confidence (confirmed.HC) hits had mean value for triplicate wells significantly different (*P* < 0.01) from vector wells, confirmed hits mean was within 99% confidence interval for positive control SNAI2. **B.** All confirmed hits grouped by functional category. Full functional annotation and clustering is presented in Supplementary Tables S3 and S4. **C.** Network plot representing known protein-protein interactions between hits (yellow nodes) and known EMT markers (green nodes). Pink nodes represent known EMT markers that were hits in the secondary screen. The lines represent STRING database interaction scores of above 0.4, obtained from either experimental evidence including homolog interaction in other species (black) or inferred from text-mining and co-expression data (grey lines). The layout was optimised for clarity of interactions between hits and EMT markers. For simplicity we omitted interactions between the EMT markers themselves as well as the unconnected nodes from either the hits or the interrogated EMT markers (TCF4).

Putative hits were re-screened in triplicate wells with freshly produced virus originating from sequence-verified expression vector clones (Supplementary Table S2). We also included the ORF coding for VIM which, surprisingly, failed hit criteria in the primary screen. Based on VIM antibody fluorescence measurements (well mean total intensity, and/or proportion of VIM positive cells, Figure 3), 14 genes had mean values significantly different (*p* < 0.01, Tukey HSD test) from empty vector controls and were classified as high-confidence confirmed hits (confirmed.HC, Figure 3A). An additional 34 genes had mean values within the 99.9% confidence interval of the positive control *SNAI2* and at least 2 out of 3 wells outside the same interval for vector, and were classified as confirmed hits (Figure 3A). Sequence analysis confirmed that all ORFs coded for full length protein except for *MAP3K11, MYOZ2,* and *WP2NL* which were truncated. Of the 60 hits that were selected based on reporter scores in the primary screen, two (*ANKRD36BPI* and *KLF3*) were confirmed by antibody score in the secondary screen. In both the primary and the secondary screen, the proportion of VIM positive cells in hit wells varied. This is most likely a reflection of the dynamic nature of EMT, so that at the time of fixation only a proportion of cells retained both attachment to the plate (epithelial feature) and VIM expression (mesenchymal feature).

Full functional annotation and clustering of these 48 genes using DAVID [23] is presented in Tables S3 and S4, while a summary of the main functional classes is shown in Figure 3B. Only four of hits (*SNAI1, SNAI2, NFKB1* and *MAP3K7*, indicated by * in Figure 3B) have been previously included in lists of EMT- promoting genes by recent meta-analyses [4, 24, 25]. The largest category of genes represented within validated hits coded for transcription factors (Figure 3B), and they were also significantly enriched (UP_KEYWORDS: Transcription regulation, 2.74 fold enriched, *P* = 7.9E-04, Table S4). Other functional categories represented included cell cycle-related genes (*CCNF, PSMB4, TRIP13, WBP2NL, YWHAE, YWHAG*), genes coding for signalling proteins including 5 membrane receptors (*EDA2R, FCGR1A, FCGR2B, LTBR, TNFRSF12A*) and three mitogen-activated protein kinases (*MAP3K11, MAP3K7, MAP3K9*), as well as cytoskeleton (*KRTAP12-3, MYOZ2, SORBS2, VIM*) and extracellular matrix (*SPOCK2*) proteins (Figure 3B). Among the hits, two signalling pathways were significantly enriched: the MAP kinase pathway (BIOCARTA: h_mapkPathway, 11.7 fold enriched, *P* = 2.36E-04) and the tumour necrosis factor pathway (GOTERM_BP_DIRECT: GO:0033209, 14.8 fold enriched *P* = 3.16E-04)(Table S4). Interrogation of the STRING protein-protein interaction database [26] for association between hits and known epithelial (*CDH1*) and mesenchymal (*CDH2, VIM*) markers or EMT associated transcription regulators (*SNAI1, SNAI2, SOX9, TCF3, TCF4, TWIST1, ZEB1, ZEB2*), resulted in a small number of experimentally demonstrated interactions (black lines, Figure 3C). There was a slightly higher number of interactions inferred from text-mining and co-expression data (Figure 3C, grey lines). In total, only 11 of the hits (not counting *VIM, SNAI1,* and *SNAI2*) had known interactions with the EMT markers. Together these analyses indicate that the majority of our hits are newly discovered drivers of EMT.

### Expression of hits in patient-derived tissue samples

**Figure 4:**
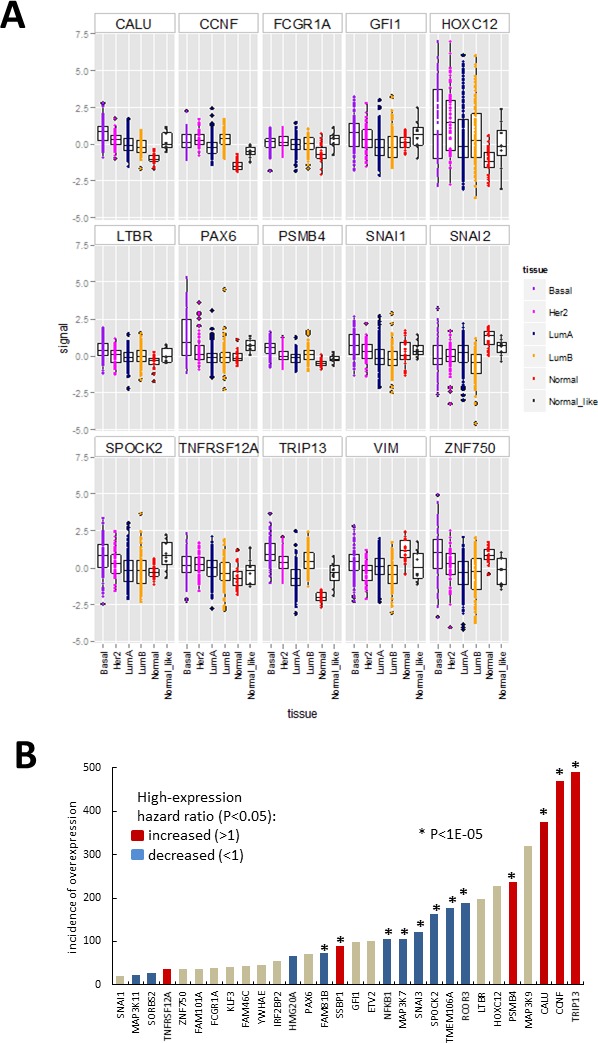
Hit gene expression in the 547 sample breast carcinoma TCGA microarray data-set (https://tcga-data.nci.nih.gov/docs/publications/brca_2012/) **A.** Boxplots (median, 25^th^ and 75^th^ quantile) overlayed with scatterplot of normalised expression (signal = log ratio) of hits differentially expressed between normal samples and/or tumour categories (indicated by point colour) assigned using clinical features and PAM50 gene signature. Boxplots for additional genes are shown in Supplementary Figure S3. **B.** Data summary for Figures 4A, S3 and S4. Incidence of tumour samples with expression signal above the maximum observed for normal breast tissue in the above-data set, with bar colour summarizing results from Kaplan-Meier survival plots (Supplementary Figure S4) obtain using the Kaplan-Meier Plotter [28]. Patient data was divided based on median level expression for each gene and data from all treatment groups and cancer types pooled.

One caveat of gain-of-function screening is that the resultant hits may not necessarily be expressed in relevant tissue *in vivo*. To identify hits that are expressed in breast cancer, we investigated publically available gene expression data derived from breast tumours. We used the TCGA data-set containing 525 tumour-derived samples and 22 normal tissue controls, classified using PAM50 profiles into molecular subtypes: basal, luminal A, luminal B, HER2, normal-like and normal [27]. We extracted the data for our hits as well as some known EMT drivers (Figure 4, Supplementary Figure S3). All of the hits had detectable transcripts in most of the samples except for *ANKRD36BP1, EIF1AD* and *ENKUR*. When the expression levels of hits (Figure 4A, Supplementary Figure S3A) and EMT marker controls (Supplementary Figure S3B) were compared across tumour subtypes, several of the hits (Figure 4A) had higher median level of expression in some tumour subtypes compared to normal tissue. In particular basal tumours, which are associated with increased EMT, had higher median levels of *CALU, CCNF, FCGR1A, HOXC12, GFI1, LTBR, PSMB4, PAX6, SNAI1, SPOCK2, TNFRSF12A, TRIP13,* and *ZNF750* (Figure 4A). The expression levels of *SNAI2* and *VIM* varied across the TCGA sample set, with median levels generally lower in tumour samples compared to control (Figure 4A). In summary, thirty of the hits were expressed at higher levels than the maximum observed for normal, in at least 20 and up to 490 of the investigated tumour samples (Figure 4B). For these thirty hits, we investigated association between high gene expression and patient survival by calculating hazard ratio using the web tool Kaplan-Meier Plotter [28] (Figure 4B, Supplementary Figure S4). The analysis was performed using microarray data and median expression level as a cut-off, and the patients were not stratified by cancer subtype or treatment. High levels of *TRIP13, CCNF, CALU, PSMB4* and *SSBP1* were strongly associated with decreased patient survival (*p* < 1E-5). In contrast, high levels of *RCOR3, TMEM106A, NFKB1, SPOCK2, SNAI3, MAP3K7* and *FAM81B* were associated with increased survival (Figure 4B, Supplementary Figure S4). At the lower confidence P levels (1.E-03 to 0.05), *TNFRSF12A* showed increased hazard ratios while *HMG20A*, *SORBS2* and *MAP3K11* decreased hazard ratios. It should be noted that this analysis does not differentiate between functional transcripts and transcripts containing function-altering mutations, and therefore the directionality of association is inconclusive. Also since the patients were not stratified, the associations that may be relevant to a disease subtype or stage were not detected, as illustrated by the lack of effect of controls *SNAI1* and *SNAI2* (Supplementary Figure S4), which are known to be prognostic in metastatic disease [4]. Nevertheless, even this conservative analysis indicated that the hits are differentially expressed in breast cancer and that some of them may influence disease *in vivo*.

**Figure 5:**
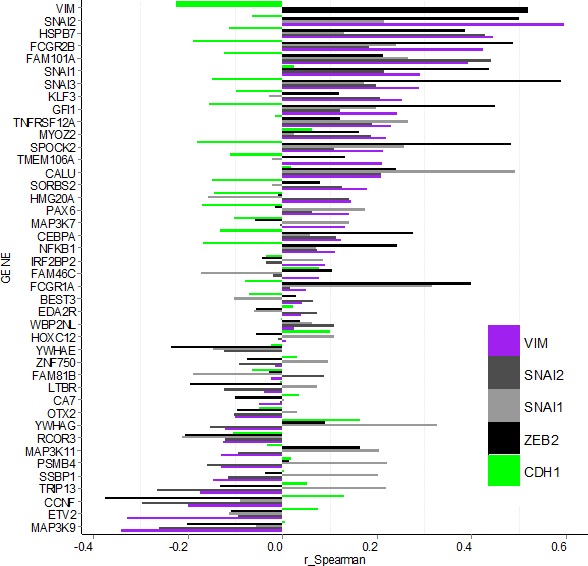
Bargraph for Spearman correlation coefficients (r_Spearman) for gene expression between confirmed hits and select EMT markers in the TCGA breast carcinoma data-set Hit-genes (y-axis) are ordered according the level of correlation with mesenchymal marker VIM (purple). Correlation with epithelial marker *CDH1* is indicated by the green bars, while the EMT drivers *SNAI1, SNAI2* and *ZEB2* are shown in grey and black. Data for additional EMT markers is shown in Supplementary Figure S4 and Table S5.

Next we investigated potential correlation between expression of hits and known EMT markers in the TCGA dataset by calculating the Spearman correlation coefficient for all gene pairs (Figure 5, Supplementary Table S5 and Figure S5). Of the hits, only *MAP3K9* and *ETV2* had negative or no correlation with both the mesenchymal marker *VIM* and any of the EMT driving factors (*SNAI1, SNAI2, SOX9, TCF3, TCF4, TWIST1, ZEB1* and *ZEB2*). Nineteen of the hits had a positive correlation (r > 0.1) with *VIM* (*NFKB1, CEBPA, MAP3K7, PAX6, HMG20A, SORBS2, CALU, TMEM106A, SPOCK2, MYOZ2, TNFRSF12A, GFI1, KLF3, SNAI3, SNAI1, FAM101A, FCGR2B, HSPB7* and *SNAI2*). They were also positively correlated with one or more of the EMT drivers, and negatively or not correlated with the epithelial marker *CDH1*. Although for some of the individual combinations correlation values were low and varied in degree of significance (see P-values in Supplementary Table S5), the overall pattern suggests that these hits may contribute to EMT in tumours *in vivo*. Expression level of *FCGR1A*, although not correlated with *VIM* or *CDH1*, had r = 0.31 and r = 0.42 with *SNAI1* and *SNAI3* respectively, suggesting it may be co-expressed with these EMT drivers in some tumours. Contrary to their observed effect on VIM protein in our study, *CCNF, TRIP13, SSBP1, PSMB4, MAP3K11, RCOR3* and *YWHAG* were negatively correlated with *VIM* (r < −0.1) at transcript level *in vivo*. At the same time except for *RCOR3* and *MAP3K11*, expression of all of these hits had a positive correlation with *TCF3* and/or *SNAI1*. Between hits themselves, the highest positive correlation (r > 0.42) was among *SNAI3, SPOCK2, GFI1, FCGR2B* and *FCGR1A*, with *GFI1* and *SPOCK2* having highest value (r = 0.81), followed by *SNAI3* and *SPOCK2* (r = 0.69).

### Functional analysis of hits in non-tumorigenic cell line MCF10A

To further examine the capability of hits to induce an EMT-like phenotype, we used the spontaneously immortalised breast-epithelial cell line MCF10A. This cell line is dependent on growth factors (EGF and insulin) for proliferation and is non-tumorigenic in mouse models [29]. Importantly, it has been shown to undergo EMT in response to TGFβ [30] and low cell density [8, 31]. Conversely, these cells exhibit an epithelial phenotype when cultured at near confluence, so we used high cell densities in all assays described below.

**Figure 6:**
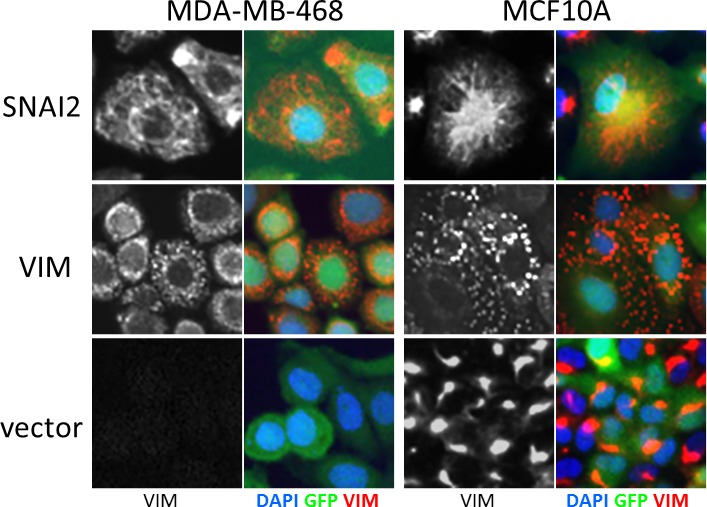
Effect of control virus on VIM fibre morphology in MCF10A and MDA-MB-468 cells *SNAI2* overexpression induces spreading of VIM into a cytoplasmic network, while VIM overexpression induces accumulation in inclusion bodies in both cell lines. Level of VIM in empty vector treated cells is higher in MCF10A cells compared to MDA-MB-468. VIM antibody (black and white) and pseudo-coloured overlay of all three channels (blue-DAPI, green-GFP, red-VIM), field width = 82.5 μm).

**Figure 7:**
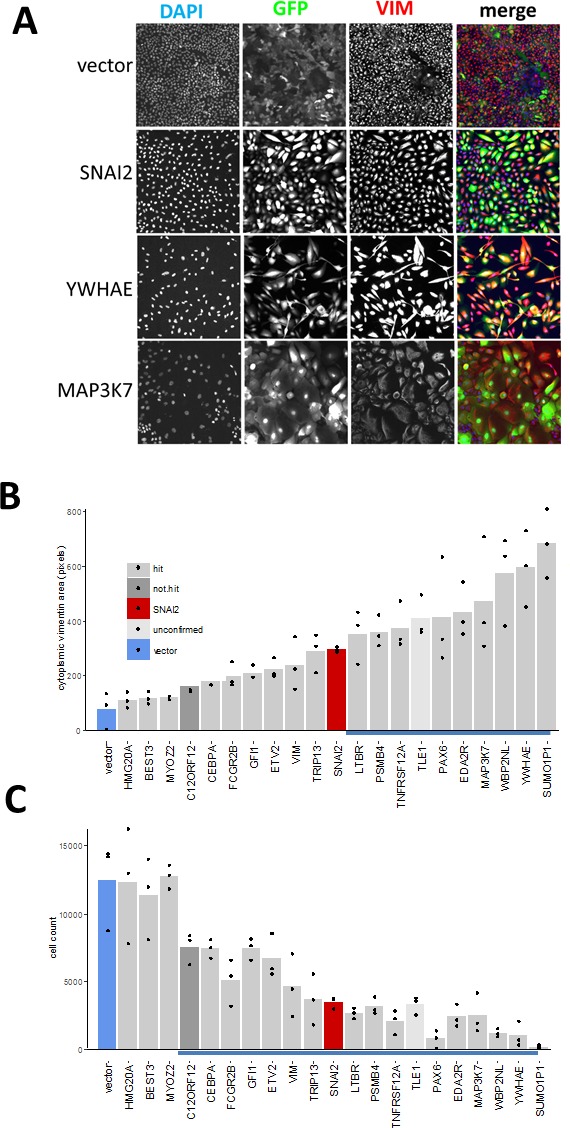
Effect of hits on VIM expression and cell morphology in MCF10A cells **A.** Individual channels and pseudo-coloured overlay of all three channels (blue-DAPI, green-GFP, red-VIM antibody). All images represent one field of view **C.** width 1321 μm). **B. C.** Bar graphs of corresponding quantitative image analysis indicating mean VIM positive cell area and cell numbers respectively, in wells transduced with indicated hit virus. Only GFP positive cells were included in calculation of VIM positive area. Bars represent mean and points represent individual values for 3 wells (20 fields were collected for each well), blue underlined bars are significantly different from vector (*P* < 0.05, ANOVA, Tukey HSD).

First, we assayed the effect of hits on VIM levels in MCF10A cells six days after transduction using high-content image analysis. We selected 18 hits that induced highest levels of VIM in the secondary screen, and included two (*C12ORF12* and *TLE1*) that failed hit criteria during primary or secondary screening respectively. Unlike the MDA-MB-468 cells, which had undetectable levels of VIM, the MCF10A cells contained cytoplasmic VIM patches when transduced with vector alone (Figure 6). Overexpression of *SNAI2*, which induces VIM as well as other mesenchymal factors, caused VIM to spread into a filamentous network in both cell lines. Interestingly, in both cell lines *VIM* overexpression induced formation of cytoplasmic VIM granules reminiscent of inclusion bodies (Figure 6). In the MCF10A cells, most of the hits tested induced formation of VIM filament networks of varying shape (Figure 7A), accompanied by increase in mean VIM area (Figure 7B). An increase in mean VIM area was observed with all hits, and was statistically significant (*p* > 0.05, Tukey HSD) for 10 of the hits. This increase in VIM area also increased the cell area so that cells remained near confluent, despite the concomitant reduction in adherent cell number (Figure 7C), which was rarely observed in the MDA-MB-468 cells.

**Figure 8:**
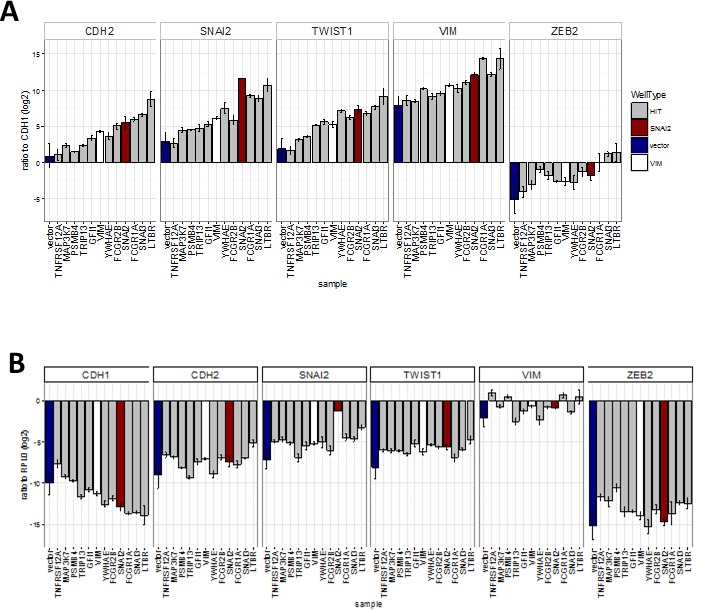
Effect of hits on EMT marker expression obtained by qRT-PCR analysis of cell-lines stably expressing hit genes (sample) (Bar = mean, error bar = SD, *n* = 3 technical replicates) **A.** Ratio of transcript levels for mesenchymal markers (*CDH2, SNAI2, TWIST1, VIM* and *ZEB2*) and epithelial marker (CDH1). All were significantly (*P* < 0.05, ANOVA, Tukey HSD) different from vector in two separate experiments except for: *TNFRSF12* all ratios, *MAP3K7 - CDH2* ratio*; and PSMB4-VIM* ratio, **B.** Ratio to ribosomal protein RPL0 control. Significantly (*P* < 0.05, ANOVA, Tukey HSD) different from vector*: CDH1* ratio *- LTBR, FCGR1A* and *SNAI3; CDH2* ratio *- LTBR; SNAI2* ratio *- LTBR* and *SNAI2; TWIST1* ratio - all ORFs; *ZEB2* ratio - *MAP3K7* and *TNFRSF12A*.

Next we investigated whether the hits were capable of inducing changes in EMT marker mRNA expression, as suggested by the observed correlation between expression levels of EMT markers and some of the hits in tumour samples. We successfully generated stable cell lines (13 out of 18 attempted) using hits that allowed maintenance of a high proportion of GFP-expressing as well as an adherent phenotype during continuous passaging. This criterion excluded some genes (eg. *PAX6, SUMO1P1*), as their expression could not be maintained at high level during long-term passaging. We then assayed RNA levels of EMT markers in extracts from cells harvested at confluency (Figure 8). Overexpression of most of the hits increased the mRNA ratios between mesenchymal markers (*CDH2, VIM, SNAI2, TWIST1, ZEB2*) and the epithelial marker *CDH1* (Figure 8A). This effect was most pronounced with *LTBR, SNAI3* and *FCGR1A*, which had effects greater than those of the positive control *SNAI2* on the levels of all transcripts tested, except for *SNAI2. FCGR2B, YWHAE,* and *GFI1* had smaller but still significant effects. Except for *YWHAE*, overexpression of these genes induced this effect by both decreasing the levels of *CDH1* and increasing levels of mesenchymal markers when compared to control transcript for the ribosomal protein RPLP0 (Figure 8B). *YWHAE* and *TRIP13* strongly decreased *CDH1*, but had a lesser effect on mesenchymal markers (Figure 8B). *MAP3K7* and *PSMB4* did not affect *CDH1* transcript levels and *TNFRSF12A* actually increased them, resulting in a smaller overall effect, despite increasing levels of all mesenchymal markers tested (Figure 8B).

**Figure 9:**
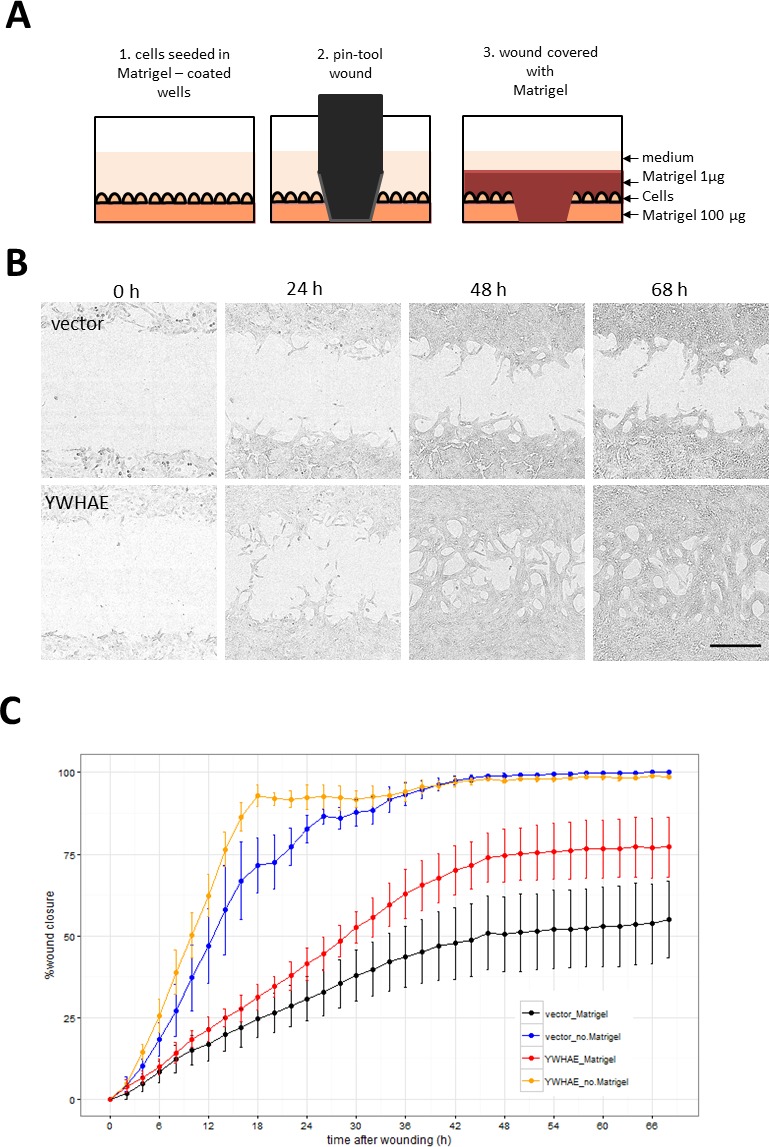
Assay for cell invasion through extracellular matrix using Matrigel™-filled scratch-wound **A.** Diagram illustrating steps in the assay set up resulting in the 3D layering of cells between 2 layers of Matrigel™. Images were collected by Incucyte every 2 h so that both rate and the degree of wound invasion can be measured. **B.** Incucyte images showing cells invading through Matrigel™ in YWHAE but not in vector treated cells (Bar = 300 μm). **C.** Comparison of wound closure profiles for traditional 2D migration assay (without the Matrigel™) and the invasion assay (with Matrigel™) for vector and YWHAE - stably transduced cells (Point = mean, error bar = SD, *n* = 6 replicate wells).

**Figure 10:**
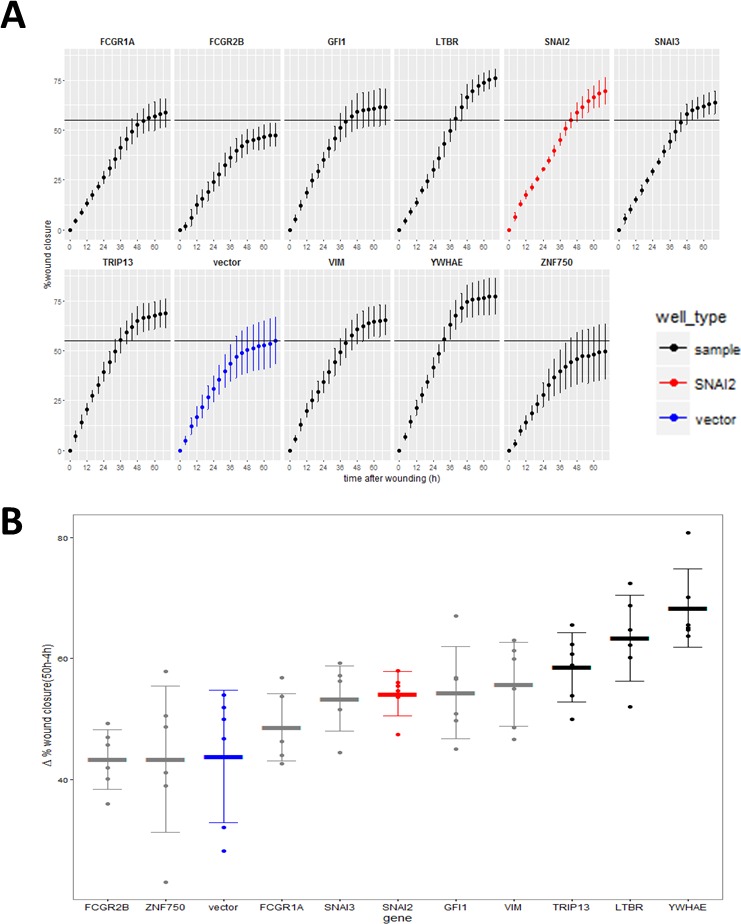
Effect of the validated hits on MCF10A cell line invasion through Matrigel™ **A.** Wound invasion profiles for hits over the 68 h observation period (red-positive control, SNAI2; blue - negative control vector). Horizontal black line indicates final mean wound closure for empty vector control (blue) (Point = mean, error bar = SD, *n* = 6 replicate wells). **B.** Wound closure rate measured between 50 h (time point when cells stopped increasing closure) and 4 h after scratching (to compensate for any possible difference in initial wound size). (Thick line = mean, error bar = SD, points = individual rate for each of 6 replicate wells). Black = genes significantly different from vector (*P* < 0.05, Tukey HSD).

Hits were further tested in a functional assay (Figure 9A) for increased invasiveness, another hallmark of EMT. To distinguish between 2D migration, which epithelial cells are capable of, and invasion through extracellular matrix which is a feature of the mesenchymal phenotype, we seeded the stably-transduced MCF10A cell lines into plates coated with Matrigel^™^, a biomatrix rich in basement membrane components designed to mimic extracellular matrix *in vitro*. After 24 h the cell layer was scratched and another layer of Matrigel™ deposited on top so that the remaining cells were trapped in this 3D matrix (Figure 9A). The rate of wound healing was monitored using the automated Incucyte system which acquired images (Figure 9B) and scratch-wound measurements over a period of 68h (Figure 9C). Addition of the two Matrigel™ layers ensured that the assay measures invasion, as opposed to 2D wound-healing migration. This was confirmed by wound-closure profiles (Figure 9C), where in the absence of Matrigel™, both vector and *YWHAE* - expressing MCF10A cells completely closed the wound, though at slightly different rates. In contrast, in presence of Matrigel™, only the cells expressing the hit *YWHAE* spanned the whole wound (Figure 9B, 9C). Of the hits tested in this assay (Figure 10), all except for cells treated with vector, *FCGR2B* and *ZNF750*, invaded the whole wound from edge to edge. Wound-closure profiles (Figure 10A) indicated that *FCGR1A, GFI1, LTBR, SNAI2, SNAI3, TRIP13, VIM, YWHAE* appeared to increase both the rate of closure and the mean wound area covered compared to vector though there was high variability between replicate wells. The rate of closure in the first 50h (before media depletion in some wells) was significantly (*p* < 0.05, Tukey HSD) different for *YWHAE, LTBR,* and *TRIP13* (Figure 10B) and the final wound closure at 68h time point was significantly larger for *YWHAE, LTBR, SNAI2* and *TRIP13*.

## DISCUSSION

This is the first report of a gain-of-function genome screen assaying capability of individual human genes to induce VIM expression and promote EMT. Most of our hits are novel players in the cancer-related EMT landscape, demonstrating the utility of this approach despite the limitations imposed by the nature of the high-throughput assay used. The use of a fixed end-point plate assay allowed us to test a large number of genes but also excluded genes that caused rapid loss of cell adherence. Adherent cell loss was observed with *ZEB1* and *ZEB2* during assay development, and with *PAX6* and *SUMO1P1* during hit validation, and may in part explain the absence of the other known EMT driving transcription factors from the hit list. The observed high levels of variability in cell morphology and VIM levels in successive rounds of screening could be due to variable levels of transgene expression and cells being fixed at a different stage of EMT. This variability would eliminate potential hits with weaker effect, and may account for VIM-ORF failing the hit criteria in the primary screen. Therefore, the screen data could serve as a source of additional EMT drivers in expanded validation experiments. This is particularly true of the hits that activated VIM promoter without increasing VIM protein levels in our primary screen. It is possible that the cells were assayed before the increased VIM mRNA has been translated, as suggested by the two reporter hits from the primary screen that became antibody hits in the validation screen. There may also be additional signals required for the cell to translate the VIM transcript once it has accumulated, as suggested by a report of a MAP kinase-interacting kinase inhibitor which abolished increase in VIM protein, but not in VIM mRNA during EMT in MDA-MB-231 adenocarcinoma cells [32]. Conversely some signals/factors may not act on the *VIM* promoter at all but increase VIM protein post-transcriptionally, and others may affect transcription by acting on distal enhancers rather than the promoter.

We have identified 48 genes that can induce VIM protein in the absence of EGF in the breast cancer cell line MDA-MB-468, suggesting they may be capable of initiating EMT. The validated hits triggered EMT-like changes in both cell morphology and gene expression, but differed in the type and level of effect. The difference in vimentin fibre morphology was particularly evident in the MCF10A cells, which are non-tumorigenic. This diversity may have been caused by the variable levels of VIM protein induced by the different ORFs. Overexpressing the VIM ORF directly may lead to sequestration into inclusion bodies [33], effectively lowering the amount of active protein available. The punctate VIM staining observed in cells transduced with the VIM ORF in our study confirms this, and may explain why VIM performed weekly in functional assays. VIM interacts with many proteins, and is both regulated by and can regulate genes that effect cancer progression and EMT [34], so it is likely that our hits induce VIM expression through different interacting proteins further explaining the diverse cellular phenotypes observed. This is in concordance with previous reports that different signals such as hypoxia or EGF cause distinctive EMT gene expression profiles [13], and conversely, that even highly conserved EMT driving genes such as *SNAI1* and *SNAI2* may have different effects depending on the expressing cell type [35]. In the MDA-MB-468 model, of our hits only *GFI1, SNAI1* and *HOXC12* were upregulated during EGF induced EMT, while hypoxia induced *GFI1* [13]. Although it is possible that other hits were present below threshold levels, it is also likely that they are inducing the EMT like phenotype by the pathways either independent or upstream of the pathways activated by EGF or hypoxia.

Most of the identified genes were found to be expressed in human breast tumours, and some were differentially expressed between normal tissue and different molecular subtypes of breast cancer. Expression levels for nineteen of our hits were correlated to some degree with levels of most EMT markers in the breast tumours. These observations, together with the analysis of clinical outcome data from breast cancer patients support a role for these hits in breast cancer related EMT *in vivo*. The TCGA data we used to compare expression of our genes and EMT markers was obtained from whole tumours, and is affected by sample heterogeneity so that effect of genes overexpressed in a fraction of cells may be underestimated. It has been demonstrated that EMT occurring in a subpopulation of cells on tumour edge is sufficient for invasiveness and cancer spread [36, 37], so that the low level of expression and/or correlation between our genes and EMT markers is not necessarily indication of irrelevance.

Small number of our hits have been previously reported in EMT and cancer. Although they have been shown to differ in the level of effect and occurrence context [35, 38], Snail family members (*SNAI1, SNAI2, SNAI3*), have a well-established role in early stages of oncogenesis-associated EMT, mainly as transcriptional repressors of E-cadherin leading to loss of cell polarity and adhesion [39]. Like the Snail proteins, the GFI1 oncoprotein is also a member of the SNAG family of transcriptional repressors [40] and interestingly is a likely target of SNAI2 [41]. While it has been mostly studied in the context of haematopoietic tumours, there is evidence of a possible oncogenic role in lung cancer [42]. The NF-kB pathway, of which NFKB1 is a component, also has a well-documented role in promoting both oncogenesis and EMT mediated by TNF cytokine signalling [43]. TNF receptor superfamily member *TNFRSF12A* (Fn14) also interacts with this pathway, is upregulated in many cancers [44], and has recently been shown to promote EMT in human bronchial epithelium [45]. *MAP3K7* is a component of the TGF-β signalling pathway that has been implicated in cancer and EMT, although its effects vary. In breast cancer models, *MAP3K7* promoted EMT and tumorigenesis [46, 47], while in other systems it was the knockdown of this gene that promoted EMT [48]. Depending on the splice isoform, calumenin (*CALU)* expression has been shown to both promote [49, 50] and inhibit [51] cell migration, so that its role in tumour metastasis may differ between cancer types [50, 52]. Reduction of Hsf1 reduces HSPB7 and inhibits EMT and tumorigenesis in mouse breast cancer models [53]. *FCGR1A* (CD64) and *FCGR2B* (CD32) code for high affinity immunoglobulin receptors found on the surface of macrophages and other immune system cells. The mechanism and significance of their ability to induce an EMT-like phenotype in mammary epithelial cells is unclear, but suggests that signalling pathways triggered by these receptors may overlap with those that induce EMT. Although the role of these genes in EMT has not been previously investigated, it has been recognised that tumour infiltrating macrophages (TIMs) play important role in cancer progression and can promote EMT [54, 55] and that targeting these receptors may affect disease outcome [56]. Based on the TCGA breast cancer microarray data alone, it is not clear if the detected expression of FCGR1A and FCGR2B is actually localised exclusively in the macrophages. Our data suggest that these receptors are capable of inducing EMT in carcinoma cells themselves.

Although it is required for and promotes cell migration, overexpression of VIM is not always sufficient to trigger EMT [57]. We have further validated some of our hits in functional EMT assays in the non-tumorigenic MCF10A cells, where they were shown to trigger EMT-like changes in cell morphology and gene expression profile. Some also increased cell invasiveness through Matrigel. This subset included genes whose expression was not strongly correlated with EMT markers *in vivo*, *YWHAE, LTBR, PSMB4* and *TRIP13*. Overexpression of *YWHAE*, a 14-3-3 protein, has been shown to promote EMT and increase invasiveness of hepatocellular carcinoma cell lines [58]. Proteosomal subunit beta 4 (*PSMB4*), has demonstrated oncogenic potential [59, 60] and can activate NFKB1 [61]. Lymphotoxin-beta receptor (*LTBR* /TNFR Superfamily member 3, *TNF3*) also activates the NF-kB pathway [62] and has been implicated in development of malignancies [63]. Thyroid hormone receptor interacting protein 13 (*TRIP13*) is upregulated in some cancers including breast cancer [64, 65]. Although it is considered to be oncogenic by promoting chromosome instability and de-activating the mitotic cell cycle check-point [66], it may have other functions. *TRIP13* knockdown reduced motility in MDA-MB-231 cells [67] and, as we have shown, *TRIP13* overexpression can promote invasion. A strong increase in invasive capacity was also observed in cells over-expressing *LTBR* and *YWHAE* and to a lesser degree *SNAI3* and *GFI1*, indicating that all these genes can drive EMT in more than one model system and have the potential to promote invasiveness of carcinomas.

In conclusion, our lentiviral overexpression screen has identified novel drivers of an EMT phenotype, including some that may have clinical relevance and others that may provide novel insights into pathway components and mechanisms involved in the process of EMT.

## MATERIALS AND METHODS

Unless otherwise indicated reagents used were from Sigma-Aldrich.

### Viral supernatant library and plasmids

Lentiviral ORF expression library arrayed in 96-well plates was obtained from and screened at the ARVEC facility at UQ Diamantina Institute [17]. Lentiviral expression constructs were generated in plvEIG (accession KF486506.1), a Gateway destination vector that allows EF1α promoter-driven co-expression of an upstream ORF and a downstream GFP, separated by an intervening IRES sequence. Control wells on each plate were: four wells containing negative control supernatant derived from plvEIG (empty expression plasmid no ORF and no ccdB gene; [18]), two “mock” wells containing viral particles without the expression plasmids, and two positive control wells expressing SNAI2.

To generate additional plates for hit validation, expression clones were re-arrayed from bacterial glycerol stocks, DNA isolated, and fresh virus generated by packaging in HEK293T cells as described previously [17].

### Cells and culture conditions

MDA-MB-468 VIMp-dsRED (St. Vincent's Institute, Melbourne, Australia) cell line contained dsRed fluorescent protein gene under the control of vimentin promoter [12] and was grown in DMEM, 10% foetal bovine serum (FBS), 2 mM L-glutamine and 100 U/100 μg/ml penicillin (pen) / streptomycin (strep) (Invitrogen). MCF-10A cells (ATCC) were maintained in DMEM/F12 (1:1; Invitrogen) supplemented with 5% (v/v) heat-inactivated horse serum (Invitrogen), 10 μg/ml insulin, 20 ng/ml EGF, 0.5 μg/ml hydrocortisone (Bayer), 100 ng/ml cholera toxin, and 100 U/100 μg/ml pen/strep. HEK293T cells (Broad Institute, Cambridge MA) were maintained in DMEM supplemented with 10% (v/v) heat-inactivated FBS (Hyclone), 0.85 mM HEPES, 2 mM L-glutamine, 1 mM sodium pyruvate, 1X non-essential amino acids (GIBCO).

Stably transduced cell lines were generated by expanding cells transduced in 96 or 12-well plates and verified to be minimum 98% GFP positive by high-content imaging. When sufficient cell numbers were obtained (usually after 2-3 weeks of passaging) cells were seeded for validation experiments.

### High-throughput transductions and plate processing for imaging

Cell seeding and bulk-dispensing of medium and fixative were performed using a WellMate microplate dispenser (Thermo Fisher Scientific, Hudson, NH, USA). An ELx405 Microplate Washer (BioTek Instruments, Winooski, VT, USA) was used for post-fixation phosphate buffered saline (PBS) washes. All other liquid handling steps were performed using SciClone ALH3000 robotic workstations (Caliper Life Sciences; Hopkinton, MA, USA).

MDA-MB-460 VIMp-dsRED cells were seeded into ViewPlate96-Black plates (PerkinElmer, Waltham, MA, USA) at 1000 cells per well in a volume of 120 μl per well, and incubated overnight. The next day, media was aspirated leaving 20 μl per well. Viral supernatant containing 12 μg/ml polybrene was transferred from the library into screening plates at 30 μl per well. After 2 h incubation, 120 μl per well media was added, and plates incubated overnight. The next day media was aspirated leaving 20 μl per well, and topped up with 150 μl per well fresh media. After an additional 5 day incubation, media was aspirated and replaced with 180 μl per well 3.7 % formaldehyde in PBS. After 15 min incubation at room temperature, wells were washed by streaming 700 μl per well PBS. Unless they were processed immediately, fixed cells were stored at 4°C in 75 μl per well PBS. Fixed cells were permeabilised by dispensing 75 μl per well 0.1 % (v/v) Triton X-100 in PBS and incubating for 15 minutes. Cells were then washed in PBS and then incubated for at least one hour at room temperature in 150 μl per well blocking buffer (1.5 % bovine serum albumin (Amresco) and 0.1 % Tween20 in PBS). Blocking buffer was aspirated (residual volume 20 μl) and primary antibody (monoclonal mouse anti-human vimentin, Clone 9, M0725 (Dako)) added in 10 μl per well at 3x concentration (1:200 in blocking buffer). Plates were incubated for minimum 1 h at room temperature or overnight at 4°C. Wells were washed in PBS and then incubated with secondary fluorescently-tagged antibodies (Alexa647 conjugated goat anti-mouse IgG, A21236, Invitrogen, 1:1500 in blocking buffer) as above. After another PBS wash, plates were stained with 300 nM 4′,6-diamidino-2-phenylindole; DAPI in PBS for 1.5 h. Wells were washed, and the cells imaged in 75 μl per well PBS.

### Imaging assay

Plates were scanned using the ArrayScan VTI HCS Reader (Thermo Scientific, Rockford, IL, USA) coupled to a Twister II Plate Handler (PerkinElmer, Waltham, MA, USA). Fluorescent images were captured using a 10 x objective and the XF2046 (400-485-558-640QBDR) quadband dichroic and excitation/emission filter set (Omega, Brattleboro, VT, USA). Images were acquired and processed using the Cellomics CellHealthProfiling.v3 algorithm. Numerical parameters were collected in four filter channels: 1) DAPI- used to define nuclear mask and count objects, 2) GFP - used to select transduced cells 3) Alexa647- anti- vimentin antibody detection 4) dsRed-vimentin promoter-reporter detection. To assay total cytoplasmic vimentin, nuclear mask from Channel 1 was extended to the mask of the neighbouring cell in channels 3 and 4. The fluorescence intensity threshold was then set to select the area containing vimentin marker (antibody in Channel 3 or reporter in Channel 4). All thresholds were set using mock-transduced wells processed as above but with primary antibodies omitted. The Cellomics ArrayScan software collected per cell average and total fluorescence intensity in each channel and calculated well summaries. This data was exported and further processed in R system for statistical computation and graphics (http://www.r-project.org/). For the primary screen analysis, plate summary statistics (median and median absolute deviation (mad)) were generated using data for sample wells containing more than 100 GFP positive cells, and used to generate robust Z-scores for all wells. Hits were selected using Z-scores and raw values as described in the main text. For subsequent validation screen, replicate wells were treated as independent samples and means compared using ANOVA and posthoc Tukey's honest significant difference (Tukey HSD) test.

### Predicted protein-protein association analysis

Predicted association between hit-ORFs and EMT marker genes was performed using STRING.v 10 (http://string-db.org/) [26]. Experimental and predicted association data with combined score > 0.4, was extracted and network figure generated in R.

### Gene expression data analysis

The 547-sample breast-carcinoma expression data set was obtained from The Cancer Genome Atlas Network (https://tcga-data.nci.nih.gov/docs/publications/brca_2012/ [27]). Expression values for hit genes and controls were extracted and processed in R. Correlation matrices were generated by calculating correlation values (r) using the Spearman method, which does not require linearity and is not sensitive to outliers.

### Invasion assay

ImageLock™ 96-well microplates (Essen Bioscience) were pre-coated with 50 μl per well Matrigel^™^ (BD Biosciences) at 100 μg/ml. Cells were seeded at 20 000 cells per well in phenol-red free media and incubated overnight. Wounds were created using a WoundMaker™ (Essen Bioscience), a 96-pin mechanical device designed to create homogeneous, 700-800 micron-wide scratch wounds in cell monolayers. After scratching, debris was removed by aspirating and dispensing fresh media at least two times using a manual multichannel pipette. Cells and the wound were then covered with media containing 1 μg/ml Matrigel™. Plates were incubated and live cell imaging was performed using an IncuCyte ZOOM (Essen Bioscience). Wound closure was quantified using the relative wound density metric by the instrument software, at 2 h intervals for the next 68 h. For the control migration assay, Matrigel™ was omitted and after wounding, cells were covered with media alone.

### RNA extraction and quantitative real-time PCR

Total RNA was isolated by harvesting cells in TRIzol^®^ and purified using the PureLink^®^ RNA Mini Kit (Thermo Fisher Scientific, Waltham, MA). RNA was quantified by NanoDrop^®^ (Thermo Fisher Scientific). Complementary DNA was synthesised from 1 μg of total RNA using the Tetro cDNA Synthesis Kit (Bioline, Taunton, MA). Pre-designed KiCqStart^®^ SYBR Green oligonucleotide primer pairs (Sigma-Aldrich, St. Louis, MO) used were: H_SNAI2_1, H_CDH1_1, H_CDH2_2, H_TWIST1_1, H_ ZEB2_1, H_VIM_1, and H_GAPDH_2 or H_RPLP0_1 as reference genes. Quantitative real-time PCR was performed using diluted cDNA with a SensiFAST SYBR^®^ Lo-ROX Kit (Bioline). Gene expression analysis reactions were observed with the ViiATM 7 Real-Time PCR System (Thermo Fisher Scientific). The run method included denaturing at 95°C for 10 seconds, annealing at 60°C for 30 seconds, and elongation at 72°C for 20 seconds. All KiCqStart^®^ SYBR Green primers had an amplicon size between 75 and 200 base pairs and were validated *in silico* with a designed computational PCR by Sigma-Aldrich.

## SUPPLEMENTARY MATERIAL FIGURES AND TABLES












