# Supplementary material for: Genome-wide gain-of-function screen for genes that induce epithelial-to-mesenchymal transition in breast cancer
Source: Oncotarget. 2016 Aug 16;7(38):61000–20. doi: 10.18632/oncotarget.11314 (PMC5308632; doi:10.18632/oncotarget.11314)
Supplement: Supplementary file 1 [file oncotarget-07-61000-s001.pdf]

Genome-wide gain-of-function screen for genes that induce epithelial-to-mesenchymal transition in breast cancer

Supplementary Material

Supplementary Table S1 – Primary screen data

Supplementary Table S2 – Secondary screen data

Supplementary Table S3 – Functional annotation of confirmed hits

Supplementary Table S4 - Functional clustering of confirmed hits

Supplementary Table S5 – Spearman correlation coefficients and P-values for microarray gene-expression data for hits and EMT controls in breast carcinoma samples from TCGA.

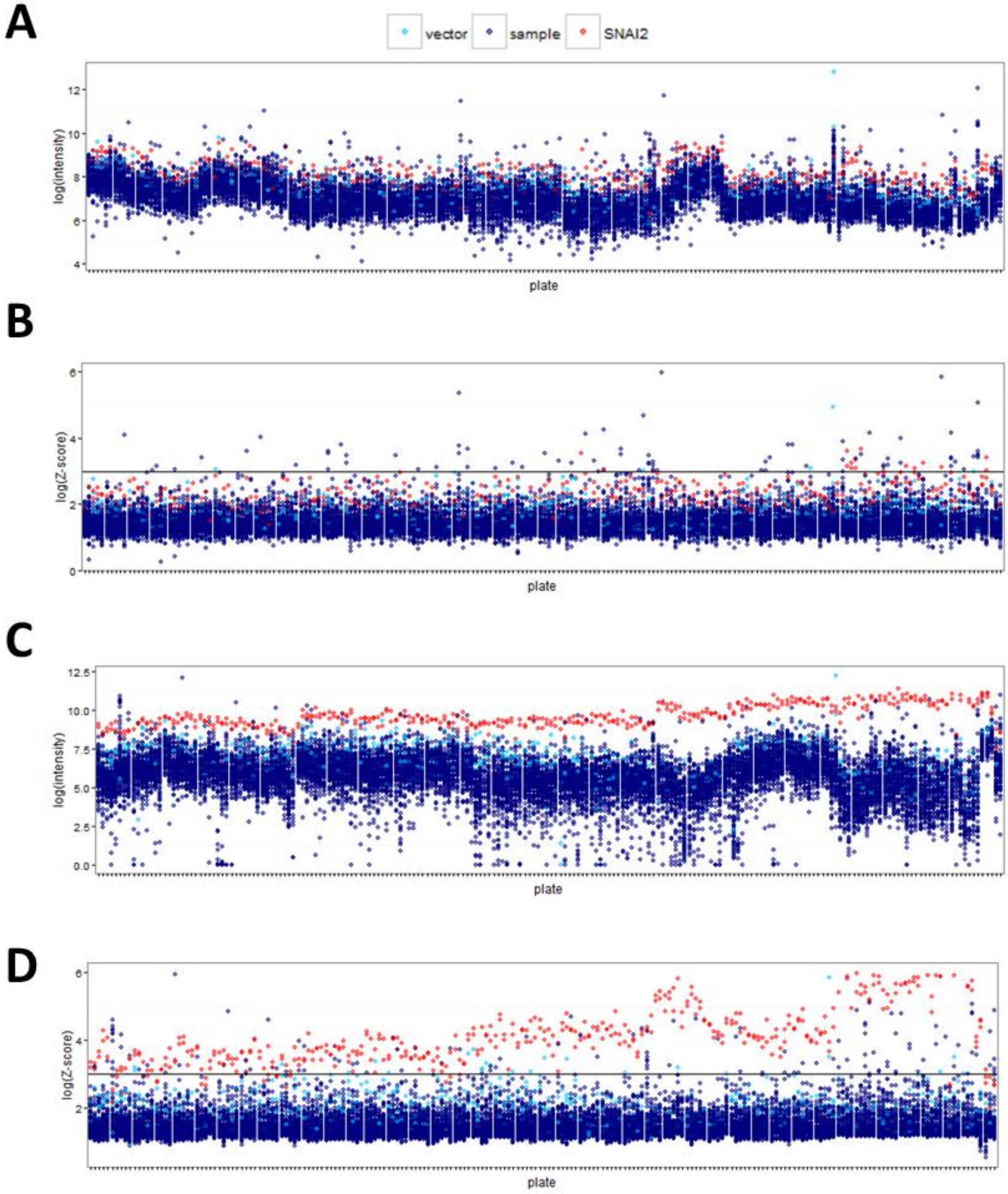

Supplementary Figure S1. Statistical analysis of screen data. Raw intensity (A and C) and normalised (robust Z-score, B, D) primary screen data for VIM fluorescence (y-axis, log2 transformed) assayed in reporter (A, B) and antibody (C, D) channels. Points in the scatterplot are grouped by plate (x-axis). Data was normalised using plate median values for wells with more than 100 GFP positive cells. Negative

control wells (vector, n=4 per plate) were used as normalisation quality control, as the supernatant for the negative controls was generated during library construction. Negative control wells were used to set thresholds for hit selection. Supernatant for the positive control (*SNAI2*, n=2 per plate) virus was generated independently of the library in several batches. Post-normalisation variation in positive control signal was used as indicator of variability in potential hits, so that although there are clear trends in the SNAI2 signal, they were not compensated for during analysis. Thresholds for hit-picking (black line) during the primary screen were set to allow 4.6 % false positive/negative rate.

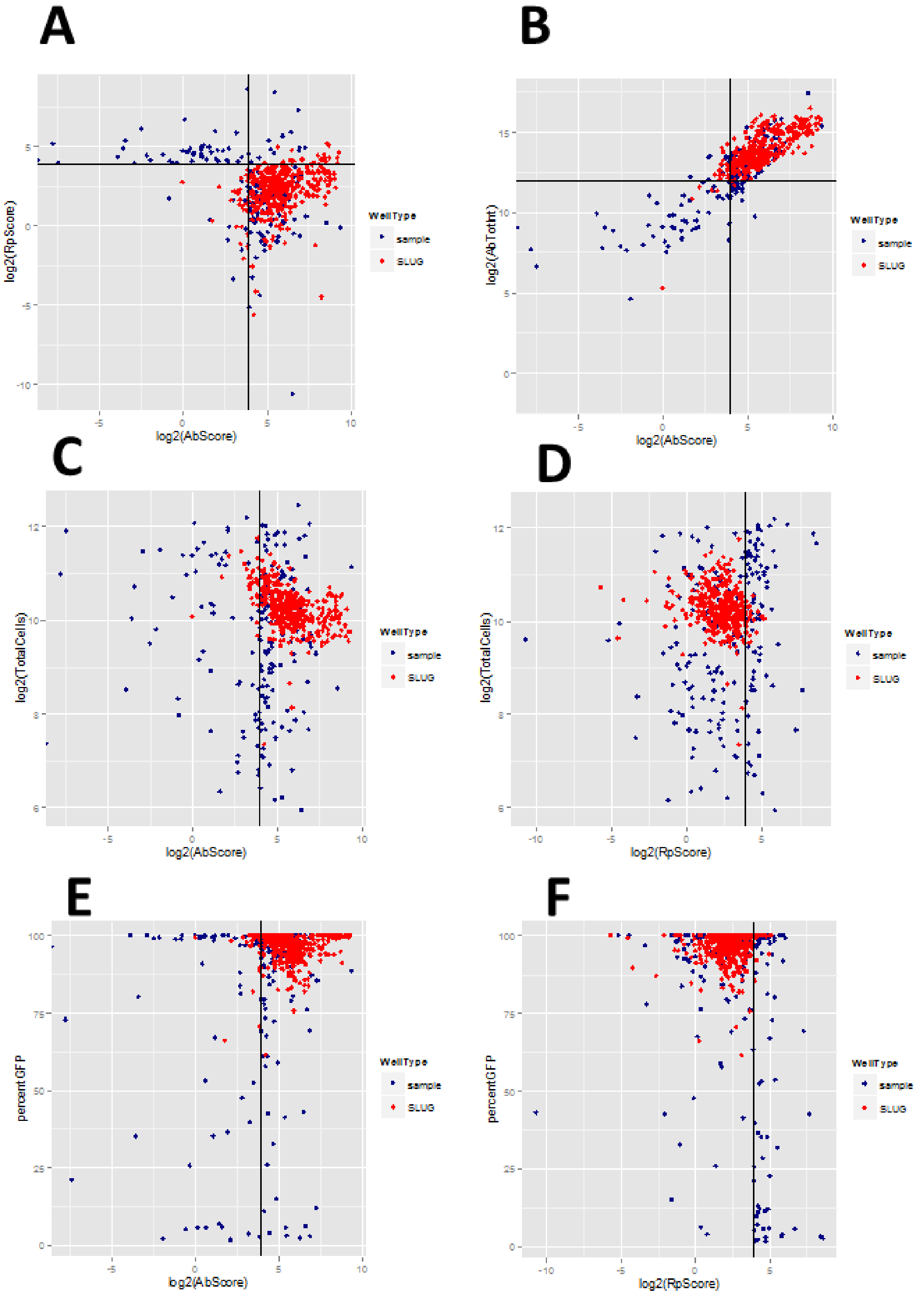
A
log2(RpScore)
log2(AbScore)
WellType
sample
SLUG
B
log2(AbTotInt)
log2(AbScore)
WellType
sample
SLUG
C
log2(TotalCells)
log2(AbScore)
WellType
sample
SLUG
D
log2(TotalCells)
log2(RpScore)
WellType
sample
SLUG
E
percentGFP
log2(AbScore)
WellType
sample
SLUG
F
percentGFP
log2(RpScore)
WellType
sample
SLUG

Supplementary Figure S2. There is no correlation between any of the variables (cell number, antibody score, reporter score and transduction rate) assayed in individual channels. Scatterplots comparing component variables between selected primary hits (navy) and SNAI2 positive control (red) wells: (A) x=antibody Z-score, y=reporter Z-score; (B) x=antibody Z-score, y= percent VIM.a positive cells; (C) x=antibody Z-score, y=total cells; (D) x=reporter Z-score, y=total cells; (E) x=antibody Z-score, y=percent GFP positive cells; (F) x=reporter Z-score, y=percent GFP positive cells.

A

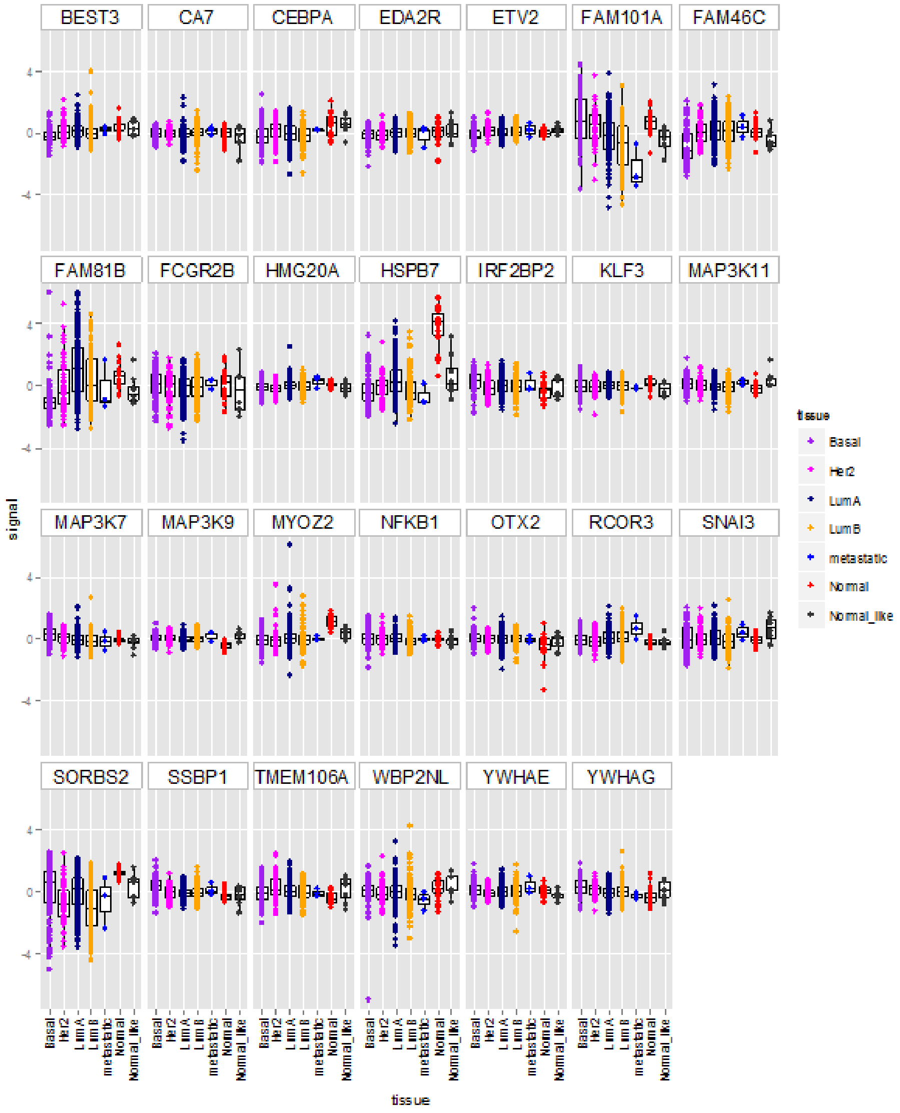
BEST3
CA7
CEBPA
EDA2R
ETV2
FAM101A
FAM46C
FAM81B
FCGR2B
HMG20A
HSPB7
IRF2BP2
KLF3
MAP3K11
MAP3K7
MAP3K9
MYOZ2
NFKB1
OTX2
RCOR3
SNAI3
SORBS2
SSBP1
TMEM106A
WBP2NL
YWHAE
YWHAG
signal
tissue
tissue
Basal
Her2
LumA
LumB
metastatic
Normal
Normal_like

B

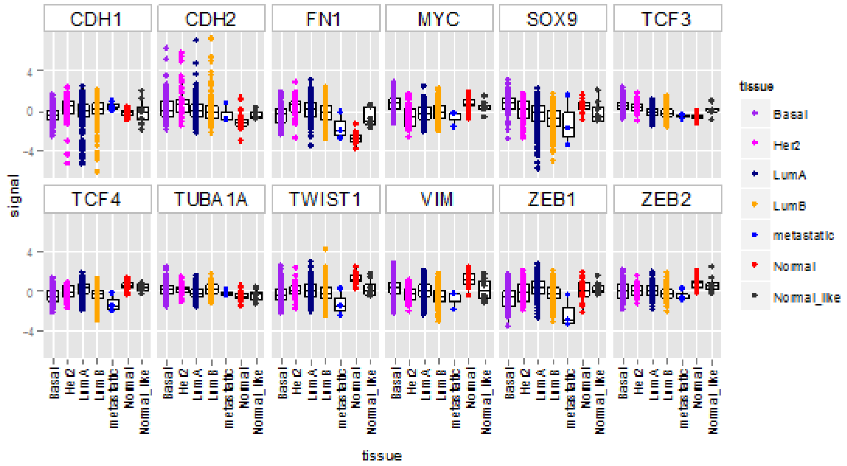
CDH1
CDH2
FN1
MYC
SOX9
TCF3
TCF4
TUBA1A
TWIST1
VIM
ZEB1
ZEB2
signal
tissue
tissue
Basal
Her2
LumA
LumB
metastatic
Normal
Normal_like

Supplementary Figure S3. Boxplots (mean, 25th and 75th quantile) overlayed with actual normalised gene expression values from the breast carcinoma data-set from the TCGA. Expression of hits (A) and select EMT markers (B) is compared across PAM50 tumour subtypes.

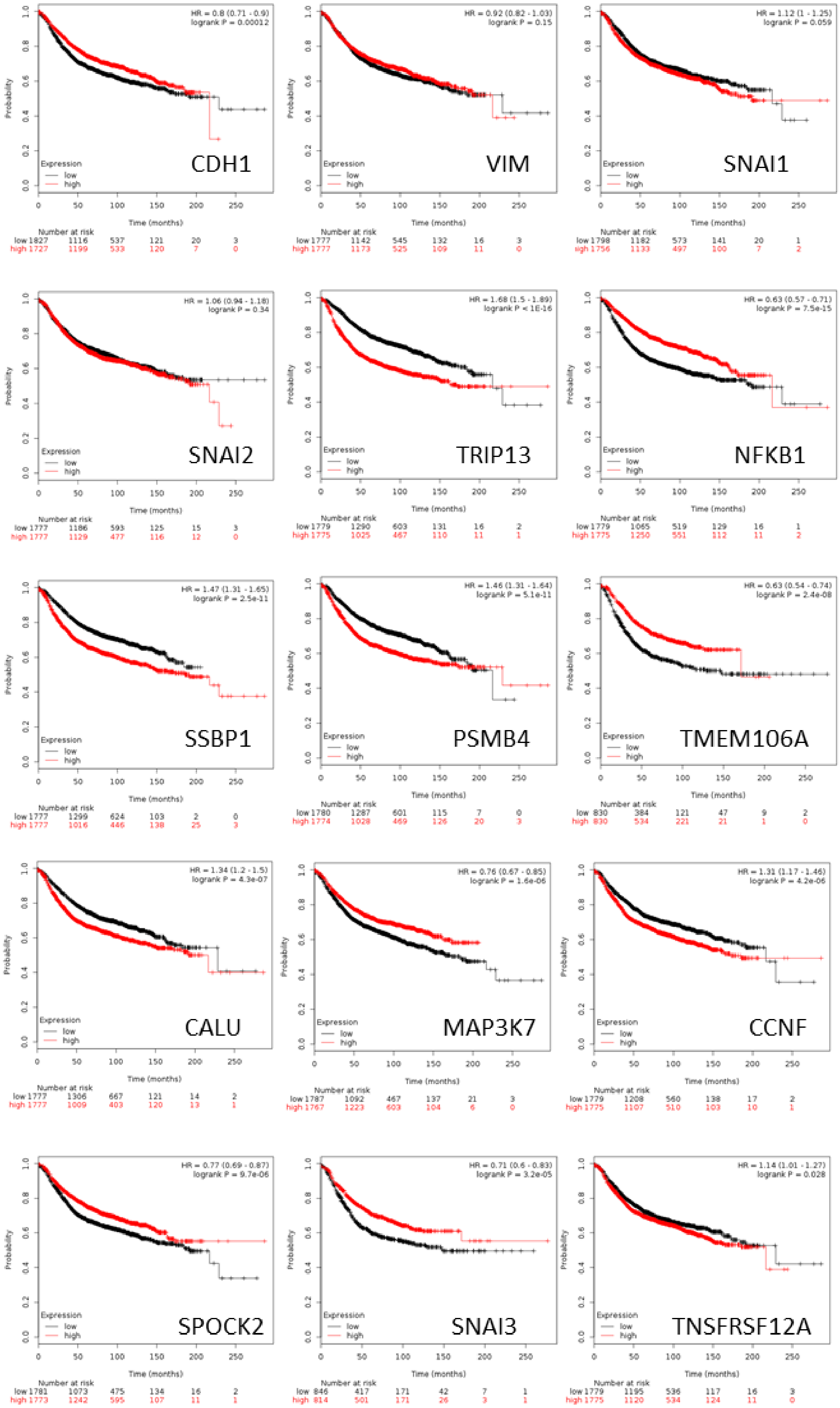
HR = 0.8 (0.71 - 0.9)
logrank P = 0.00012
CDH1
HR = 0.92 (0.82 - 1.03)
logrank P = 0.15
VIM
HR = 1.12 (1 - 1.25)
logrank P = 0.059
SNAI1
HR = 1.06 (0.94 - 1.18)
logrank P = 0.34
SNAI2
HR = 1.68 (1.5 - 1.89)
logrank P < 1E-16
TRIP13
HR = 0.63 (0.57 - 0.71)
logrank P = 7.5e-15
NFKB1
HR = 1.47 (1.31 - 1.65)
logrank P = 2.5e-11
SSBP1
HR = 1.46 (1.31 - 1.64)
logrank P = 5.1e-11
PSMB4
HR = 0.63 (0.54 - 0.74)
logrank P = 2.4e-08
TMEM106A
HR = 1.34 (1.2 - 1.5)
logrank P = 4.3e-07
CALU
HR = 0.76 (0.67 - 0.85)
logrank P = 1.6e-06
MAP3K7
HR = 1.31 (1.17 - 1.46)
logrank P = 4.2e-06
CCNF
HR = 0.77 (0.69 - 0.87)
logrank P = 9.7e-06
SPOCK2
HR = 0.71 (0.6 - 0.83)
logrank P = 3.2e-05
SNAI3
HR = 1.14 (1.01 - 1.27)
logrank P = 0.028
TNSFRSF12A
Expression
low
high
Probability
Time (months)
Number at risk

Supplementary Figure S4. Kaplan-Meier survival plots for select hit genes over-expressed in breast cancer. Panels are ordered from top-left showing EMT controls (CDH1, VIM, SNAI1 and SNAI2) first, followed by the hits ordered by the increase in unadjusted P-value. Data was obtained and graphed using the KM Plotter [28]. Patients were split into high and low expression groups using the automatically determined best median cut-off for values from JetSet best probe set for each gene. Analysis was not restricted by disease subtype or patient cohort.

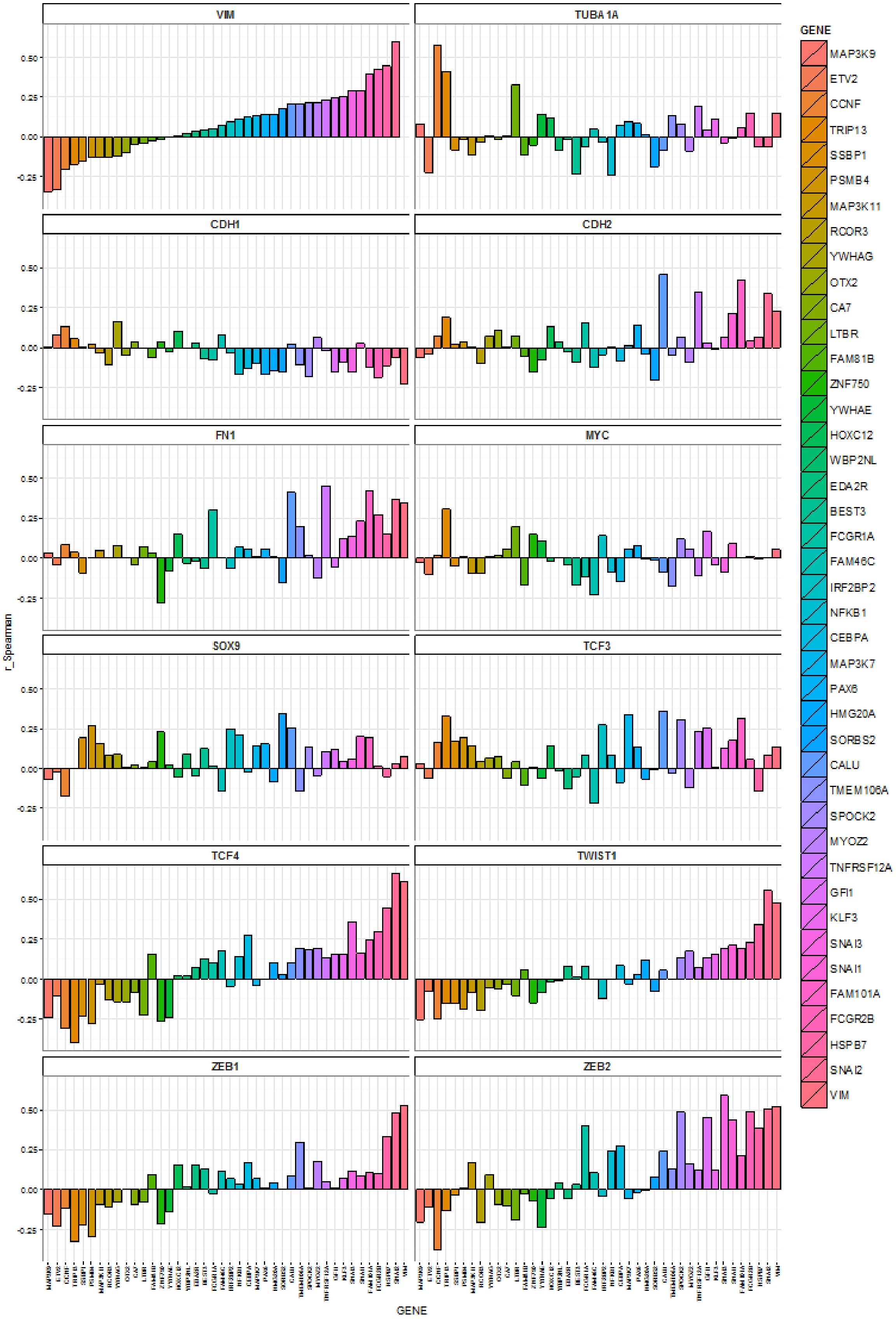
VIM
TUBA1A
CDH1
CDH2
FN1
MYC
SOX9
TCF3
TCF4
TWIST1
ZEB1
ZEB2
0.50
0.25
0.00
-0.25
r_Spearman
GENE
GENE
MAP3K9
ETV2
CCNF
TRIP13
SSBP1
PSMB4
MAP3K11
RCOR3
YWHAG
OTX2
CA7
LTBR
FAM81B
ZNF750
YWHAE
HOXC12
WBP2NL
EDA2R
BEST3
FCGR1A
FAM46C
IRF2BP2
NFKB1
CEBPA
MAP3K7
PAX6
HMG20A
SORBS2
CALU
TMEM106A
SPOCK2
MYOZ2
TNFRSF12A
GFI1
KLF3
SNAI3
SNAI1
FAM101A
FCGR2B
HSPB7
SNAI2
VIM

Supplementary Figure S5. Bar graphs for Spearman correlation coefficients (r_Spearman) for gene expression between confirmed hits and select EMT markers (facets) in the TCGA breast carcinoma data-set. Hit-genes (x-axis) are ordered according the level of correlation with VIM. Bars are coloured for ease of comparison, for P-values see TableS5.
